# Which Strength Qualities Matter Most for Sprinting? Partitioning Unique and Shared Variance in a Multivariable Model

**DOI:** 10.1002/ejsc.70216

**Published:** 2026-06-27

**Authors:** Luke R. Stutter, David L. Carey, Minh Huynh, Matthew W. Driller, Charlie J. Davids, Lachlan P. James

**Affiliations:** ^1^ Sport, Performance, and Nutrition Research Group School of Allied Health, Human Services, & Sport La Trobe University Melbourne Victoria Australia

**Keywords:** athlete, performance, physical preparation, resistance training, sprinting

## Abstract

Individual strength qualities have been associated with sprint performance, but previous researchers have not partitioned the unique and shared variance each contributes, nor included the full range of strength qualities in a single analysis. This study is the first to model all five strength qualities (heavy dynamic, fast dynamic, reactive, isometric, and explosive) concurrently against sprint performance. Multiple regression was used to quantify the unique and shared variance each quality contributes across multiple sprint distances. Eighty‐four resistance‐trained individuals (62 male, 22 female; 21.4 ± 3.4 years, 79.9 ± 12.1 kg), completed countermovement jumps, drop jumps, isometric squat, isometric mid‐thigh pull, and a three‐repetition maximum (3RM) back squat, alongside a 40‐m sprint. Three multiple linear regression models were constructed to partition the unique and shared variance contributed by each strength quality to sprint performance across 5–40 m and maximal velocity, with sex included as a covariate. Combining countermovement jump height, 3RM back squat, and drop jump reactive strength index explained up to 75.2% of variance in sprint performance. The addition of isometric peak force or force at 100 milliseconds resulted in negligible increases in explained variance.

## Highlights

- Fast dynamic strength, operationalised via CMJ height, is the most notable strength quality to explain sprint performance when accounting for each of the five distinct strength qualities in a multiple regression.
- Heavy dynamic (3RM back squat) and reactive (drop jump RSI) strength measures contributed significantly within the sprint models, but isometric and explosive strength qualities did not.
- A three‐test model (CMJ height, 3RM back squat, drop jump RSI) explained up to 75.2% of variance in sprint performance.
- Practitioners should prioritise fast dynamic, heavy dynamic, and reactive strength in training and assessment.

## 1 Introduction

Sprinting is a pivotal skill within many sports, as faster players are better able to cover ground quickly, change direction abruptly, and outrun opponents, ultimately impacting game outcomes (Misseldine et al. 2021). Speed has been linked to greater athletic success, with faster individuals more likely to be drafted (Edwards et al. 2021) or reach elite levels within their sport (Baker and Newton 2008). As a result, the physical qualities underpinning sprinting are of high importance in athletic development. Force production capabilities are multi‐dimensional. Acceleration is determined by the magnitude of net horizontal force produced relative to body mass, with greater relative force enabling larger changes in velocity per step (Nagahara et al. 2018). Critically, as sprint velocity increases, ground contact times shorten progressively, reducing the time available for force application and shifting the demand from force magnitude to rate of force development and reactive capabilities. This time‐constrained nature of force application provides the mechanistic basis for considering distinct strength qualities.

While strength is defined as the ability to produce force (Stone 1993), a contemporary framework describes five broad and distinct force production characteristics: heavy dynamic, fast dynamic, reactive, isometric, and explosive (James et al. 2023). The framework is generalised, and the complexity of underlying neuromuscular qualities may be reflected in population‐specific differences (Geneau et al. 2024; Geneau, Carey, et al. 2025; Geneau, Gastin, et al. 2025). Terminology within the strength and conditioning literature remains inconsistent, with terms such as ‘explosive’, ‘reactive’, and ‘power’ often used interchangeably or defined by different temporal or mechanical thresholds (Winter et al. 2016). Importantly, several metrics used to operationalise these domains, such as CMJ jump height, drop jump RSI, and ground contact time, represent downstream products or expressions of force production characteristics rather than direct measures of strength qualities (Geneau et al. 2024; Geneau, Carey, et al. 2025). Nevertheless, we retain the framework here because it represents the most comprehensive contemporary structure for partitioning lower‐body strength qualities, but acknowledge these caveats when interpreting model outputs. This study defines explosive capabilities as force production achieved within ≤ 150 ms, consistent with previous literature (James et al. 2023). Measures of each strength quality have been shown to influence sprint performance at different stages throughout a sprint (de Villarreal et al. 2012; Jarvis et al. 2022; Seitz et al. 2014), producing either large horizontal propulsive forces (Morin et al. 2015) when accelerating or high vertical forces under time constraint (Paradisis et al. 2019) during maximal velocity sprinting. However, there is a lack of comprehensive analysis that partitions the unique versus shared variance each strength quality contributes to sprint performance across its distinct phases. This is important for practitioners when determining which qualities to target, and which are deemphasised due to redundancy, to enhance speed.

The assessment of strength qualities to inform sprint performance (Cronin and Hansen 2005; Young et al. 1995) has been dominated by analysing a single variable and a sprint performance outcome. Greater levels of heavy dynamic (Barr and Nolte 2011; Hori et al. 2008; Wisløff et al. 2004), isometric (Brady et al. 2020; Townsend et al. 2019), explosive (Brady et al. 2020; Tillin et al. 2013), fast dynamic (Barr and Nolte 2011; Furlong et al. 2019), and reactive (Brady et al. 2024; Möck et al. 2021) qualities have been associated with superior acceleration or maximal velocity sprint performance. Whilst correlation analyses provided useful preliminary insight, they are inherently limited and do not consider the common variance shared with other variables. Specifically, when a range of strength qualities and associated measures are of interest, univariate approaches cannot determine whether an association with sprint performance is truly unique or merely reflective of variance it shares with other candidate measures (James et al. 2023). Furthermore, correlation analyses do not infer cause‐and‐effect relationships, limiting their ability to identify the mechanisms underpinning performance. Strength qualities may also interact, suppress, or mediate one another's effects (James et al. 2023). Accounting for these relationships can change the magnitude of an effect by removing irrelevant variance. Without doing so, correlation studies risk misrepresenting the true predictive nature of any single quality. While causal insights are better established through well‐controlled training interventions, appropriately specified multivariate models provide a framework for understanding the unique and combined contributions of physical qualities to sprint performance.

Multi‐variable analyses provide deeper understanding of the combined effects each individual strength quality has on sprint performance. For example, relative squat performance did not significantly correlate with 30‐m sprint time when analysed in isolation, despite showing a non‐significant moderate association (std. *β* = 0.459). However, it emerged as one of the strongest predictors when modelled alongside jump variables (Furlong et al. 2019). Conversely, multiple metrics from squat, drop and countermovement jump variations showed moderate‐large correlations to a range of sprint distances (*r* = 0.421–0.683), yet were reduced to either squat jump height or reactive strength index when modelled via a multiple regression (Smirniotou et al. 2008). A multi‐variable approach is therefore required to model the range of specific strength qualities concurrently, partitioning the unique and shared variance contributed by each and identifying which matter most for sprint performance.

Whilst multi‐variable analyses have been utilised previously to explain sprint performance (Brady et al. 2024; Furlong et al. 2019; Northeast et al. 2019; Smirniotou et al. 2008; Wijekulasuriya et al. 2024), several limitations have been identified. Each study was restricted by small sample sizes (15–26 individuals) and did not cover the full spectrum of strength qualities. For example, heavy dynamic (Furlong et al. 2019; Wijekulasuriya et al. 2024) and isometric strength qualities (Brady et al. 2024) were utilised in independent studies, but have not been included together despite evidence the two are not interchangeable qualities (James et al. 2024). Greater levels of each strength‐related qualities were concluded to enhance sprint performance, however, it is not clear whether this reflects a shared underlying neuromuscular construct (e.g., muscle mass, voluntary activation) or whether each contributes distinctively due to biomechanical or temporal specificity (Suchomel et al. 2018). Resolving this requires concurrent modelling of all five qualities to determine which are less important, and which offer unique value for assessment and training. To address these gaps, the current study uses multiple regression to model all five strength qualities against sprint performance across multiple distances, quantifying the unique and shared variance each strength quality contributes. The aim of the study was to clarify their relative importance for sprint performance and inform more targeted assessment and training. It was hypothesised that heavy and fast dynamic strength would uniquely explain the most variance during acceleration and that fast dynamic and reactive strength would explain the most variance during maximal velocity sprinting.

## 2 Methods

### 2.1 Design

A cross‐sectional study design was implemented to (i) determine the extent to which combinations of measures across all strength qualities could best explain sprint performance, and (ii) which of these measures and associated qualities contributed the greatest to model classification. Testing involved a series of jump, isometric and heavy loaded tests, as well as sprint performance. A ten‐minute warm up was performed which included dynamic stretching, mobility, sub‐maximal jump and sprint efforts. A sample‐size was calculated using G*Power for a medium effect size (*f* ^2^ = 0.15), based on Cohen's conventions (Cohen 1988) and consistent with previous literature reporting small‐to‐moderate associations between strength qualities and sprint performance (Furlong et al. 2019; Smirniotou et al. 2008), with alpha set at 0.05 and power at 0.80 which estimated 49 participants. Multicollinearity was assessed for all models using variance inflation factors.

### 2.2 Participants

Recruitment involved mostly tier 2 and some tier 3 athletes (McKay et al. 2022) (*n* = 84 [62 male, 22 female]; 21.4 ± 3.4 years; 79.9 ± 12.1 kg) who participate in field sports (Australian football, soccer, hockey, etc.). They were required to have a minimum of 12 months resistance training experience, proficiency in the back squat exercise, and free of injury or illness. Prior to testing participants underwent a pre‐exercise screening questionnaire and gave written consent. Ethical approval was obtained from the university ethics committee (HEC23220).

### 2.3 Methodology

#### 2.3.1 Sprint Assessment

Two maximal effort sprints over 60‐m were performed on a grassed surface using a standing 2‐point start, with 3 minutes rest between efforts, ensuring participants were not constrained by distance in order to reach maximal velocity. A global positioning system (GPS) device (Vector S7, Catapult Sports, Melbourne, Australia) captured speed data which is a reliable and validated tool for speed assessment (Clavel et al. 2022). A custom R script was used for analysis. Threshold detection of 0.2 ms^−1^ was used to initiate the start of the sprint and calculate time taken for splits up to 40‐m, with the fastest sprint effort included for analysis.

#### 2.3.2 Strength Qualities Assessment

The assessment of specific strength qualities included drop jump (DJ), countermovement jump (CMJ), isometric squat (ISQ), isometric mid‐thigh pull (IMTP), and a three‐repetition (3RM) back squat. Jump and isometric testing followed previous protocols (Barr and Nolte 2011; Brady et al. 2018) and were performed on force plates using Forcedecks software (VALD, Brisbane, Australia) in a custom‐made isometric frame. All tests were completed in a single session, in the order described above, deemed least‐fatiguing to most‐fatiguing, with three to 5 minutes rest between measures. Participants were familiarised with the testing protocol prior to their session.

Following a standardised warm up, participants performed three trials of the drop jump from a 45‐cm box, which represented the assessment of reactive strength. Each trial was supervised to ensure drop height was consistent. Participants were cued to jump as high as possible, with minimal contact time on the ground. Only jumps with contact times less than 250 milliseconds (Jarvis et al. 2022) were deemed acceptable for analysis, with the highest reactive strength index (RSI) score determined via jump height divided by contact time. Drop jump RSI was used to operationalise reactive strength capacity, reflecting the efficiency of force production within the temporal constraints of ground contact. Quantification of fast dynamic strength was operationalised via the CMJ test, with jump height serving as the primary outcome metric. Participants performed three trials of a maximal jump with countermovement to a self‐selected depth. The jumps with the greatest jump height (via impulse‐momentum method) were used for analysis. A wooden dowel (400 g) was held across the shoulders to remove arm involvement during both the drop jump and CMJ and standardise across trials.

To quantify isometric and explosive strength both an isometric squat and IMTP were utilized. In both tests, a 70% and 90% effort was performed prior to two maximal efforts where peak force needed to be within 250 N or a third attempt was carried out (Comfort et al. 2019). In the ISQ, the bar sat across the upper trapezius with an upright torso and knee angle of 140° used to represent the strongest position (Brady et al. 2018). A stable force output of approximately 100 N of pretension above bodyweight was held for 3 seconds prior to a maximal isometric force output (Comfort et al. 2019) in the squat movement pattern. The IMTP followed a similar protocol (Brady et al. 2018) with the inclusion of wrist straps. The test metrics included net peak force (PF_net_), relative PF_net_ (rPF_net_) and net force at 100 milliseconds (F_100_) metrics. The test effort with the highest F_100_ measure was used for analysis.

A 3RM back squat was used to measure heavy dynamic strength. The warm up included sets of eight, five and three repetitions at 50%, 70% and 85% of estimated testing weight, respectively. Participants then performed a 3RM to a depth of thigh parallel, determined by a qualified practitioner. If the subject performed the repetitions successfully, they were instructed to increase the weight with a rest period of 5 minutes between testing sets. The process was repeated until the participant could no longer perform three successful repetitions with full range of motion, or both the subject and the researcher did not think another attempt with greater load would be successful. The maximum weight lifted was used for analyses, in absolute and relative form.

### 2.4 Statistical Analyses

Modelling of a comprehensive range of strength qualities was employed to predict sprint time to distances 5–40 m and maximal velocity (*V*_max_). Multiple regression analyses were performed to construct three models for each sprint distance, using key metrics from each of the five distinct strength qualities as independent variables. Both IMTP and ISQ were included separately to determine any differences between either isometric method, as well as a simplified model using only fast dynamic, heavy dynamic and reactive strength. Sex was included as a covariate in all models. Model assumptions (e.g., normality of residuals, homogeneity of variances, multicollinearity) were assessed using the performance package in R (Version 4.3.1).

## 3 Results

Summary data of sprint and strength quality variables can be seen in Table 1. The ISQ model (Table 2) was able to explain between 48.9%–75.5% of variance in sprint performance. Similarly, the IMTP model (Table 3) explained 49.7%–77.9% and the simple model (Table 4) explained 47.9%–75.2% of variance.

**TABLE 1:** Descriptive statistics of strength and speed variables included in the analyses.

Variable	Mean	SD
DJ RSI	2.64	0.46
DJ contact time (ms)	193	25
CMJ jump height (cm)	34.79	7.14
IMTP net peak force (*N*)	2111	525
IMTP force 100 ms (*N*)	586	303
ISQ net peak force (*N*)	2226	718
ISQ force 100 ms (*N*)	550	240
Squat 3RM (kg)	108.1	27.0
Sprint 5 m (s)	1.23	0.10
Sprint 10 m (s)	2.01	0.15
Sprint 20 m (s)	3.34	0.25
Sprint 30 m (s)	4.59	0.40
Sprint 40 m (s)	5.82	0.54
Sprint *V*_max_ (m/s)	8.35	0.90

*Note:* DJ contact time is presented as a descriptive variable only and was not included as a predictor in the regression analyses.

**TABLE 2:** Modelling of strength metrics and sprint performance with isometric squat.

Predictors	Sprint_5m	Sprint_10m	Sprint_20m	Sprint_30m	Sprint_40m	Sprint_*V*_max_
	Std. *β*	Std. *β*	Std. *β*	Std. *β*	Std. *β*	Std. *β*
Squat 3RM	−0.21	−0.19	−0.21	−0.18	−0.21	0.19
	(−0.51–0.09)	(−0.46–0.08)	(−0.45–0.03)	(−0.40–0.04)	(−0.42–0.00)	(−0.02–0.39)
CMJ JH	−0.24	−0.27^*^	−0.26^*^	−0.27^**^	−0.25^**^	0.20^*^
	(−0.50–0.01)	(−0.50 to −0.04)	(−0.46 to −0.06)	(−0.45 to −0.08)	(−0.43 to −0.07)	(0.03–0.38)
DJ45 RSI	−0.12	−0.07	−0.12	−0.12	−0.14	0.16^*^
	(−0.34–0.09)	(−0.26–0.12)	(−0.29–0.05)	(−0.28–0.03)	(−0.28–0.01)	(0.01–0.30)
ISQ F100	0.08	0.08	0.04	0.05	0.05	−0.07
	(−0.10–0.26)	(−0.09–0.24)	(−0.10–0.18)	(−0.08–0.18)	(−0.08–0.17)	(−0.19–0.06)
ISQ netPF	−0.11	−0.1	−0.06	−0.03	−0.02	0.03
	(−0.36–0.14)	(−0.33–0.13)	(−0.26–0.14)	(−0.21–0.15)	(−0.20–0.15)	(−0.15–0.20)
Sex [m]	−0.46	−0.74^**^	−0.83^***^	−0.98^***^	−1.00^***^	1.12^***^
	(−0.97–0.06)	(−1.20 to −0.28)	(−1.23 to −0.42)	(−1.36 to −0.60)	(−1.36 to −0.64)	(0.76–1.47)
*R* ^2^/*R* ^2^ adjusted	0.489/0.449	0.586/0.554	0.681/0.656	0.725/0.703	0.752/0.733	0.755/0.736

^*^ *p* < 0.05.

^**^ *p* < 0.01.

^***^ *p* < 0.001.

**TABLE 3:** Modelling of strength metrics and sprint performance with isometric mid‐thigh pull.

Predictors	Sprint_5m	Sprint_10m	Sprint_20m	Sprint_30m	Sprint_40m	Sprint_*V*_max_
	Std. *β*	Std. *β*	Std. *β*	Std. *β*	Std. *β*	Std. *β*
IMTP F100	0.14	0.21^*^	0.20^**^	0.18^**^	0.18^**^	−0.19^**^
	(−0.04–0.32)	(0.05–0.37)	(0.06–0.34)	(0.05–0.31)	(0.06–0.30)	(−0.31 to −0.07)
Squat 3RM	−0.29^*^	−0.24^*^	−0.23^*^	−0.18	−0.19^*^	0.17
	(−0.55 to −0.02)	(−0.48 to −0.01)	(−0.43 to −0.03)	(−0.37–0.01)	(−0.36 to −0.01)	(−0.01–0.34)
CMJ JH	−0.25^*^	−0.29^*^	−0.29^**^	−0.29^**^	−0.27^**^	0.23^**^
	(−0.51 to −0.00)	(−0.51 to −0.07)	(−0.48 to −0.09)	(−0.47 to −0.11)	(−0.44 to −0.11)	(0.06–0.39)
DJ45 RSI	−0.13	−0.07	−0.12	−0.12	−0.13	0.15^*^
	(−0.34–0.08)	(−0.26–0.11)	(−0.28–0.05)	(−0.27–0.03)	(−0.27–0.01)	(0.01–0.29)
IMTP netPF	0.02	−0.01	−0.06	−0.04	−0.07	0.06
	(−0.24–0.28)	(−0.24–0.21)	(−0.26–0.14)	(−0.22–0.15)	(−0.24–0.11)	(−0.11–0.23)
Sex [m]	−0.54	−0.84^***^	−0.89^***^	−1.06^***^	−1.05^***^	1.18^***^
	(−1.10–0.01)	(−1.33 to −0.36)	(−1.31 to −0.47)	(−1.45 to −0.67)	(−1.42 to −0.69)	(0.81–1.54)
*R* ^2^/*R* ^2^ adjusted	0.497/0.457	0.615/0.585	0.709/0.686	0.749/0.729	0.776/0.758	0.779/0.762

^*^ *p* < 0.05.

^**^ *p* < 0.01.

^***^ *p* < 0.001.

**TABLE 4:** Simplified modelling of strength metrics and sprint performance using dynamic tests.

Predictors	Sprint_5m	Sprint_10m	Sprint_20m	Sprint_30m	Sprint_40m	Sprint_*V*_max_
	Std. *β*	Std. *β*	Std. *β*	Std. *β*	Std. *β*	Std. *β*
Squat 3RM	−0.27^*^	−0.24^*^	−0.24^*^	−0.19^*^	−0.21^*^	0.19^*^
	(−0.51 to −0.03)	(−0.45 to −0.03)	(−0.43 to −0.06)	(−0.36 to −0.02)	(−0.37 to −0.05)	(0.02–0.35)
CMJ JH	−0.23	−0.26^*^	−0.25^*^	−0.26^**^	−0.24^**^	0.20^*^
	(−0.49–0.02)	(−0.49 to −0.03)	(−0.45 to −0.06)	(−0.45 to −0.08)	(−0.42 to −0.07)	(0.02–0.37)
DJ45 RSI	−0.13	−0.08	−0.12	−0.12	−0.14	0.16^*^
	(−0.34–0.08)	(−0.27–0.11)	(−0.29–0.04)	(−0.28–0.03)	(−0.28–0.01)	(0.01–0.30)
Sex [m]	−0.45	−0.73^**^	−0.82^***^	−0.98^***^	−1.00^***^	1.11^***^
	(−0.96–0.07)	(−1.19 to −0.27)	(−1.22 to −0.42)	(−1.35 to −0.61)	(−1.35 to −0.64)	(0.76–1.47)
*R* ^2^/*R* ^2^ adjusted	0.479/0.453	0.579/0.557	0.678/0.662	0.722/0.708	0.750/0.738	0.752/0.739

^*^ *p* < 0.05.

^**^ *p* < 0.01.

^***^ *p* < 0.001.

In the ISQ modelling, CMJ jump height (as the metric operationalising fast dynamic strength) explained significant unique variance across 10–40 m (std. *β* = −0.25 to −0.27) and *V*_max_ (std. *β* = 0.20). DJ RSI was the only other metric which significantly contributed to this group of modelling, explaining *V*_max_ (std. *β* = 0.16). The remaining strength metrics did not significantly contribute to any models despite 3RM back squat suggesting a medium effect size (std. *β* = −0.18 to −0.21) for all sprint measures. ISQ F_100_ and ISQ PF_net_ coefficients were negligible (std. *β* = −0.11–0.03). For IMTP modelling, CMJ jump height, 3RM back squat and IMTP F_100_ metrics uniquely explained 5–40 m (std. *β* = −0.29–0.21). *V*_max_ was explained by significant contributions from CMJ jump height, DJ RSI and IMTP F_100_ metrics (std. *β* = −0.19–0.23), although 3RM back squat did not significantly contribute despite a medium effect size (std. *β* = 0.17). Important to note, measures of IMTP F_100_ were significant, but contributed opposingly to the other metrics.

In the simplified modelling, CMJ jump height (std. *β* = −0.26 to −0.24) uniquely explained 10–40 m and *V*_max_, 3RM back squat uniquely explained 5–40 m (std. *β* = −0.27 to −0.19) and *V*_max_ (std. *β* = 0.19), and DJ RSI significantly contributed to *V*_max_ (std. *β* = 0.16), but did not significantly explain variance in any other models (std. *β* = −0.08 to −0.14).

## 4 Discussion

The primary aim of this research was to explore which specific strength qualities, represented by a key performance metric used to operationalise the underlying strength construct, had the greatest contribution to sprint performance. Additionally, which variables were able to best explain unique variance in sprint performance to help practitioners better understand the role of strength training. It was determined that combining common performance metrics CMJ jump height, 3RM back squat and drop jump RSI explained a large amount of variance in sprint performance. Furthermore, these metrics each operationalised a distinct strength quality within the five‐quality framework, specifically fast dynamic, heavy dynamic and reactive strength respectively (James et al. 2023), and that CMJ jump height contributed most to sprint performance. In situations where time and resources are limited, practitioners should consider assessing fast dynamic, heavy dynamic and reactive strength qualities in their training practices, particularly when looking to explain variance in sprint performance.

Three models encapsulating commonly used test metrics explained similar variance in sprint performance. The variables included one commonly used metric from each strength quality including CMJ jump height (fast dynamic), 3RM back squat (heavy dynamic), drop jump RSI (reactive), net force at 100 ms (explosive) and net peak force (isometric). Models explained between 47.6%–77.9% of variance in sprint performance included sex as a covariate. 5‐m and 10‐m variance had reduced overall explained variance, however, 20–40 m and *V*_max_ had similar total explained variance, across all three models. The findings indicate that practitioners could reduce the assessable strength qualities to fast dynamic, heavy dynamic, and reactive strength, and still explain a large portion of sprint variance in their athletes.

Fast dynamic strength, operationalised by CMJ jump height, was the largest, significant predictor of sprint performance in every model from 10 to 40 m and *V*_max_. In the 5‐m distance, this measure was only significant when included alongside IMTP. Measures derived from the CMJ, and squat jump, have demonstrated moderate to strong isolated relationships with acceleration and maximal sprinting (Barr and Nolte 2011; Furlong et al. 2019), although their influence in multivariate analyses has been less commonly shown. Previous analyses have identified CMJ, or squat jump, jump height as the largest predictor of 30‐m sprint performance (Furlong et al. 2019), or as a significant contributor (Brady et al. 2024). However, limited coverage of strength qualities and small sample sizes restrict any conclusions about the unique contribution of each quality to sprint performance. Although jump height is the most commonly reported outcome metric and is used here to operationalise fast dynamic strength, it is mechanistically determined by net vertical impulse relative to body mass, which in turn reflects the magnitude and duration of force applied during the propulsive phase (McMahon et al. 2018). Jump height was retained as the primary metric due to its widespread use, practical reliability, and clear interpretability for practitioners, while still capturing the underlying impulse‐momentum relationship that defines ballistic force production. The current findings indicate that fast dynamic strength, when modelled alongside the other qualities, is the largest predictor of sprinting performance. This effect was evident across a large and varied sample, supporting the generalisability of the finding. Practitioners looking to improve sprint performances should prioritise fast dynamic strength for strength training and assessment, using ballistic tasks involving a slow stretch‐shortening cycle such as the CMJ or squat jump.

The 3RM back squat, representing heavy dynamic strength was the second largest contributor to explain sprint performance. It significantly contributed across all sprint distances in the dynamic‐only modelling, and most variables in the IMTP modelling. However, when included alongside ISQ, the 3RM back squat was not significant. Importantly, low CI values suggest further investigation could be warranted. The results of the correlation studies have shown moderate‐strong relationships with both acceleration and top‐speed sprinting (Barr and Nolte 2011; Hori et al. 2008; Wisløff et al. 2004), and suggested stronger individuals exhibit greater fast strength expression (Cormie et al. 2010). Multi‐variable analyses identified positive effects for relative squat strength (Furlong et al. 2019) and speed, and another identified a negative interaction with preseason running loads (Wijekulasuriya et al. 2024). However, no study has looked at a measure of heavy dynamic strength in combination with the remaining strength qualities. The results suggest the 3RM back squat independently explains a substantial portion of sprint performance, likely due to overlapping neuromuscular factors (Suchomel et al. 2018). These findings highlight the importance of heavy dynamic strength for both acceleration and maximal velocity sprinting.

Reactive strength plays a key role in high‐velocity sprinting due to the need for high ground reaction forces under limited contact time (Nagahara et al. 2018). Drop jump RSI, as the performance index used to operationalise reactive strength, explained a significant portion of variance in maximal velocity sprinting in the modelling. These results are aligned with previous correlation studies (Barr and Nolte 2011; Young et al. 1995) and multi‐variable analyses which have demonstrated reactive strength uniquely contribute to maximal running alongside fast dynamic (Brady et al. 2024) or heavy dynamic strength (Furlong et al. 2019). While reactive strength was not significant in the longer distance splits (30‐ and 40‐m), its contribution could potentially increase in sprint‐trained individuals who possess greater stiffness and faster top speeds than recreational athletes (Douglas et al. 2018). Despite this, reactive strength should be included in training and assessment for athletes who sprint maximally as a performance requirement of their sport.

Isometric and explosive strength did not positively influence sprint performance. Isometric strength showed no unique contribution when accounting for other strength qualities. Similarly, explosive strength (force at 100 ms) in the ISQ models was not significant at any distance, although in the IMTP models the positive beta coefficients indicated a detrimental effect on sprint variables excluding 5‐m. Previous studies found significant correlations with acceleration (Brady et al. 2020; Tillin et al. 2013; Townsend et al. 2019), and explosive strength was even shown to significantly impact 5‐m performance when modelled with fast dynamic and reactive strength (Brady et al. 2024). However, these analyses excluded heavy strength in their modelling. A substantial portion of shared variance exists between isometric and heavy dynamic strength qualities (Lum et al. 2020), as well as between explosive and heavy dynamic strength (James et al. 2023). The findings in the present study suggest force produced isometrically may not explain unique variance of sprint performance when modelled alongside other strength qualities, particularly heavy dynamic strength in the form of a 3RM squat. However, both isometric and explosive qualities can be considered in holistic strength assessment models as they may provide context to alternate strength qualities.

### 4.1 Limitations

Cross‐sectional association does not represent the long‐term effects of resistance training and sprint performance, nor individual response to training. This is compounded by the fact that domain structure may shift across time points, with population‐specific sub‐domains potentially emerging (Geneau et al. 2024, 2025). Consideration should therefore be given to regular assessment across multiple time points to monitor progression and the stability of strength qualities. Future research should investigate the long‐term training effects of fast dynamic, heavy dynamic and reactive strength qualities on sprint performance. Test order may have introduced residual fatigue. Although tasks were sequenced from least to most fatiguing with standardised rest periods and a consistent test order across participants, accumulated fatigue could not be fully avoided. Consideration should also be given to the operationalisation of strength qualities via single performance metrics. Metrics such as CMJ jump height, drop jump RSI, and ground contact time are downstream products of force production rather than direct strength measures, and reducing each domain to a scalar outcome, risks obscuring meaningful variation in force‐, velocity‐, and time‐dependent characteristics (Geneau et al. 2024, 2025). These metrics are interpreted as proxies for their respective strength qualities, consistent with the caveats noted in the introduction. Furthermore, while drop jump RSI represents the reactive strength quality, the component parts (e.g., jump height and contact time) in ratio metrics should be considered. Finally, the inclusion of sex as a categorical covariate accounts for between‐group differences, but does not fully capture the underlying physiological mechanisms driving performance differences. Emerging evidence suggests that sex differences in strength‐related performance may be largely explained by factors such as relative strength and body mass rather than sex itself (Comfort et al. 2024; Nimphius et al. 2019). Meaning its inclusion may partly reflect these underlying factors rather than representing an independent determinant of performance. Future research should consider modelling these variables directly to provide a more mechanistic interpretation of performance outcomes.

## 5 Practical Applications

Practitioners looking to implement specific strength training and assessment to enhance sprint performance in recreational and sub‐elite athletes should prioritise fast dynamic strength in their practice, followed by heavy dynamic and reactive strength. If short for time, emphasis should be placed on ballistic force production. Future research should investigate whether these findings extend to elite sprinters and sport‐specific populations, and where possible include sex‐specific analyses.

## 6 Conclusion

Fast dynamic strength was the strongest unique predictor of sprint performance when all five strength qualities were modelled concurrently, with the countermovement jump emerging as the key contributing measure. Practitioners aiming to enhance sprint performance should prioritise fast dynamic strength, followed by heavy dynamic and reactive strength.

## Funding

The authors have nothing to report.

## Ethics Statement

Ethical approval was obtained from the La Trobe University Human Research Ethics Committee (HEC23220).

## Conflicts of Interest

The authors declare no conflicts of interest.

## Data Availability

The data that support the findings of this study are available on request from the corresponding author. The data are not publicly available due to privacy or ethical restrictions.
